# Functional Balance and Its Determinants in Older People with Diabetes

**DOI:** 10.1371/journal.pone.0159339

**Published:** 2016-07-28

**Authors:** Yi-Ju Tsai, Yi-Ching Yang, Feng-Hwa Lu, Pei-Yun Lee, I-Ting Lee, Sang-I Lin

**Affiliations:** 1 Department of Physical Therapy, College of Medicine, National Cheng Kung University, Tainan, Taiwan; 2 Department of Family Medicine, College of Medicine, National Cheng Kung University, Tainan, Taiwan; 3 Department of Family Medicine, Tainan Hospital, Ministry of Health and Welfare, Tainan, Taiwan; 4 Department of Rehabilitation, Tainan Municipal An-Nan Hospital-China Medical University, Tainan, Taiwan; Harvard Medical School, UNITED STATES

## Abstract

**Objective:**

To determine functional balance abilities of older adults with diabetes, and identify determinants of these abilities.

**Methods:**

Eighty diabetic and 67 healthy non-diabetic community-dwelling older adults completed the Mini Mental Status Examination (MMSE) and questionnaires about their medical and fall histories. Participants were also assessed for vision, plantar sensitivity, muscle strength, and functional balance, including Functional Reach (FR), Five Times Sit-to-Stand (FTSTS), and 180° turn (TURN). In addition to between-group comparisons, hierarchical regression analysis was conducted to identify the independent determinants for each of the individual balance tasks for the diabetes and control group separately.

**Results:**

The diabetes group had significantly greater body mass index, higher rate of cardiac disease, and poorer plantar sensitivity, mental status, grip and lower limb strength. The diabetes group performed significantly poorer in FTSTS and TURN (both *p*<0.001), but not FR (*p* = 0.108). The significant determinants for the balance tasks varied substantially between tasks and groups. For the diabetes group, they included visual and plantar sensitivity and MMSE for FR (R^2^ = 0.39), ankle dorsiflexion strength for FTSTS (R^2^ = 0.377), and plantar sensitivity, knee extension strength and MMSE for TURN (R^2^ = 0.391). For the control group, knee extension strength emerged as the common and only significant determinant and only explained approximately 10% of the variance for FR and TURN.

**Conclusions:**

Impairments in functional balance abilities were evident for older adults with diabetes. Their underpinning functional limitations were different for different tasks and were also different from those of the control group. Screening of functional balance and mental status, lower limb strength and sensory function, and interventions to address these impairments may be important to maintain function, independence and safety for older clients with diabetes.

## Introduction

Diabetes is a major global epidemic and occurs in a quarter of older population worldwide [1,2]. The disease is particularly troubling for older adult because of its association with higher incidence of falls [3–5] and greater risk of physical disability [6–8]. Considering that imbalance is often the primary cause of falls and disability, it is probable that older adults with diabetes could have greater balance problems.

The above notion, however, is not fully supported by the limited research findings in this area. A study of inner city older African Americans showed that those with diabetes did not differ significantly in their static standing and functional balance, compared to non-diabetic healthy older adults [9]. In contrast, in a study of disabled older women, those with diabetes had significantly lower summary performance scores of walking speed and functional balance than those without diabetes [7].

The contradictory research findings are not surprising. Balance control requires complex interactions among multiple systems of the body and encompasses different balance subtasks that could be affected by pathologies or aging differently. For example, the ability to maintain postural stability during quiet standing has been found to be declined in diabetic older people with neuropathy, but not those without [10, 11]. However, the ability to regain balance after reaching forward has been shown to be reduced in older diabetic patients with or without neuropathy [12].

In addition to peripheral neuropathy, visual and somatosensory impairment, and declines in cognition that have been reported to be associated with the pathology of diabetes can also impact on effective balance performance [13]. As a result, balance problems in older adults with diabetes may be inconsistent with typical aging, and possibly have differing determinants.

Different types of balance abilities, such as moving the body’s center of mass or changing the base of support repeatedly, are often required in daily living and are critical for functional independence. It is clinically relevant to identify which types of balance abilities are affected and what their determinants are in older people with diabetes. This study thus sought to determine functional balance abilities of older adults with diabetes, and identify determinants of these functional balance abilities. This information may improve identification and effectiveness of interventions.

## Materials and Methods

Two groups of older adults were recruited to participate in this study–one group of healthy control participants, and one group of older adults with diabetes. The common inclusion criteria for both groups were being over the age of 60 years, being able to walk without person support for more than six meters, and to be able to follow three-step commands. For the control group, recruitment posters were places at the nearby community centers and the bulletin boards in the hospital where the patient group was recruited. These control participants have had their blood sugar tested to show no sign of diabetes in the past year. Diabetic participants were consecutive patients visiting outpatient clinics in the department of family medicine in a medical center. A total of 396 patients were contacted, with 314 of them declined. The diagnostic criteria for diabetes were fasting plasma glucose higher than 126 mg/dl or symptoms of hyperglycemia (polyuria, polydipsia and unexplained weight loss) and a casual plasma glucose concentration higher than 200 mg/dl [14]. The common exclusion criteria for both groups were acute inflammation or pain of the lower limbs, problems in the neuromuscular or musculoskeletal system that could affect the performance of the balance tasks, total blindness, lumbar spinal radiculopathy, and partial or total lower limb amputation. The study was approved by the Institutional Review Board of National Cheng Kung University Hospital. All study participants fully understood the protocol and risks associated with the study and provided written consent.

All participants provided basic demographic information, medical history, and fall history (self-report, within the past year), and then were assessed on the Chinese version of the Mini Mental Status Examination (MMSE, highest available score = 33) [15] and a series of vision and sensorimotor function tests. A fall was defined as an event resulting in unintentionally coming to the ground or lower level and other than as a consequence of sustaining a violent blow, loss of consciousness, sudden onset of paralysis, or an epileptic seizure [16]. Individuals who have fallen in the past year were classified as fallers. The Melbourne Edge Test was used to test visual contrast sensitivity [17] and the Snellen’s E Chart was used to test visual acuity. The Semmes Weinstein monofilament examination (SWME) was used to test plantar sensitivity [18] over three sites of each foot (the plantar surface of the metatarsal head of the first and fifth toes and the center of the heel). A JAMAR dynamometer was used to measure grip strength of the dominant hand, as an indicator of general health. A Microfet 2 handheld dynamometer was used to measure the strength of hip flexion, knee extension and ankle dorsiflexion using standardized positions and procedures [19]. Because the strength of plantarflexion cannot be reliably tested with a handheld dynamometer, the standardized manual muscle testing method was used (maximal of 25 repetitions) [19].

The functional balance tests used in this study targeted different aspects of balance control abilities and were performed in random order. For FR, a yard stick was positioned horizontally next to the right side of the participant at the height of the acromion. Participants were instructed to stand naturally, raise the right arm forward to 90° (parallel to the yard stick), and at the ‘go’ signal, reach forward as much as possible at their own pace without losing balance or moving the foot, and then return to quiet standing [20]. The test was video-taped and then viewed by an experimenter to determine the reach distance. For FTSTS, participants were asked to stand up from a 43 cm height chair and then sit back down for five repetitions as fast as possible without using the arms [21]. For TURN, the participants kept their hands by their side, and were positioned on a 40 cm x 60 cm square drawn on the ground. They were then asked to turn 180° at their own pace by taking steps within the square to face the opposite direction [22]. A digital stop watch was used to measure the time taken to complete FTSTS and TURN.

### Statistical analysis

For the comparisons between the healthy control and diabetes groups, the *chi* square tests were used for nominal variables, Mann-Whitney U tests for rank order variables and independent *t* tests for continuous variables. For the sensorimotor measurements obtained from both limbs, the averages were used for data analysis, with an exception of visual acuity for which the score of the better eye was used. Hierarchical regression analysis was used for the individual task performance for the two groups separately. For the diabetes group, four blocks were entered as the independent variable in a stepwise manner: sensory function (heel plantar sensitivity, visual contrast sensitivity), lower limb muscle strength, diabetes duration, and health conditions. A similar regression model was used for the control group, except that diabetes duration was not entered. For the lower limb strength, only those muscles having significant correlations with the task performance were entered. For the health condition, the related factors (BMI, MMSE, grip strength, hypertension, cardiac disease, and falls) that had significant correlations with the task performance were entered. The collinearity statistics of the regression models would be examined to detect if there was multicollinearity. Pearson or Spearman correlation analysis was conducted as appropriate to determine the associations between the variables. The significance level was set at *p* < 0.05. All analyses were conducted using the SPSS Statistics 20 software.

## Results

**Fig 1:**
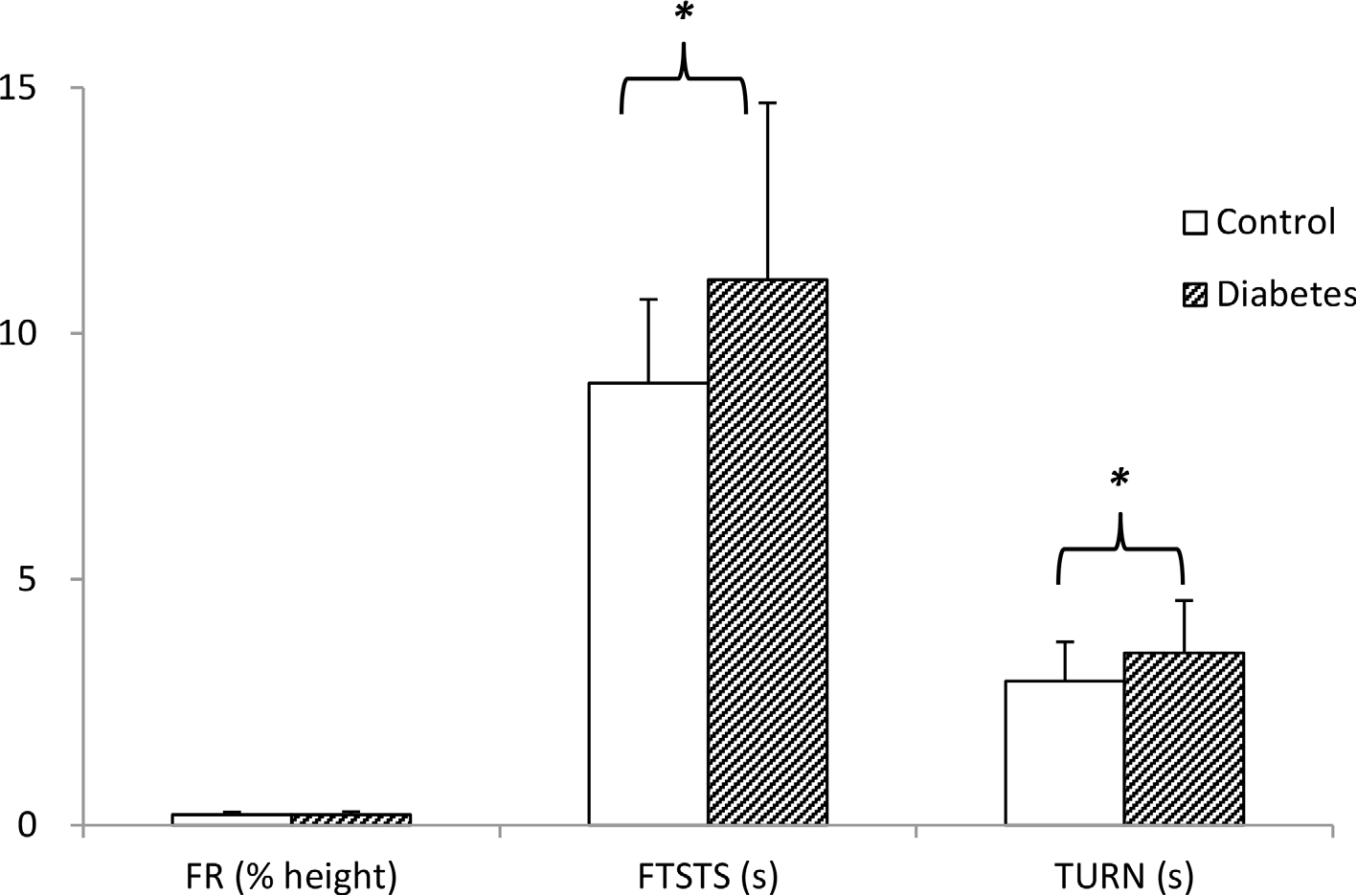
Performance of Functional Reach (FR), Five Times Sit-to-Stand (FTSTS) and Turn 180° (TURN) in control and diabetes groups. * *p* < .001

A total of 80 older adults with diabetes and 67 healthy controls completed the tests. Their basic characteristics, MMSE, diabetes complications, comorbidities, fall history and sensorimotor functions are shown in Table 1. Participants with diabetes had their condition for an average of 11 years, one third reported presence of neuropathy, and 7.5% retinopathy. Compared to the control group, the diabetes group had significantly higher body mass index and prevalence of cardiac disease, and poorer MMSE, grip and lower limb strength and big toe and heel sensitivity (Table 1). For balance performance (Fig 1), significant between-group differences were found in FTSTT (power = 0.92, *p*<0.001) and TURN (power = 0.87, *p*<0.001), but not FR (power = 0.13, *p* = 0.108).

**Table 1:** Demographic information and sensorimotor function of healthy control and diabetic participants.

	Control (n = 67)	Diabetes (n = 80)
Age (years)	68.5 (6.5)	68.7 (6.3)
Male	55.2%	56.3%
Body mass index^*^	23.3 (3.1)	25.7 (3.6)
MMSE^*^	31.1 (2.4)	30.3 (2.4)
Diabetes duration (year)	NA	11.2 (9.6)
Diabetes complications		
Neuropathy	0	33.8%
Retinopathy	0	7.5%
Nephropathy	0	1.3%
Comorbidity		
Stroke	0	1.3%
Cardiac disease^*^	9%	22.5%
Hypertension	14.9%	25%
Arthritis	11.9%	5%
Fallers	10.4%	16.0%
Vision		
Visual acuity	32.9 (19.1)	40.9 (34.7)
Visual contrast sensitivity	21.7 (2.3)	21.4(2.5)
Plantar sensitivity (log 10)		
Big toe^*^	4.19 (0.45)	4.39 (0.54)
5^th^ toe	4.28 (0.47)	4.52 (0.58)
Heel^*^	4.46 (0.51)	4.77 (0.51)
Strength (% body weight)		
Grip^*^	45.0 (9.1)	37.9 (10.1)
Hip flexion^*^	23.4 (6.2)	21.0 (5.6)
Knee extension^*^	31.5 (7.1)	28.0 (7.9)
Ankle dorsiflexion^*^	32.8 (8.2)	29.3 (6.5)
Ankle plantarflexion (repetition)^*^	11.1 (8.4)	8.0 (7.2)

NA: not available; MMSE: Mini Mental Status Examination, Chinese version (maximum score = 33)

^*^ p<0.05

### Functional reach

For the control group, FR was significantly correlated with TURN (r = -0.323, *p* = 0.008) but not FTSTT (r = -0.016, *p* = 0.897). Correlation analysis also showed that FR was significantly correlated with knee extension strength (Table 2) and these variables had low to moderate correlations with each other (absolute r = 0.114~0.416). Regression analysis showed that knee extension strength was a significant determinant and explained approximately 11% of the variance in FR (Table 3). For the diabetes group, FR was not significantly correlated with the other two tasks (r = 0.183–0.218, *p* = 0.097–0.162). Correlation analysis showed that FR was significantly correlated with ankle dorsiflexion strength and MMSE (Table 2) and these variables had low to moderate correlations with each other (absolute r = 0.026~0.365). The results of regression analysis showed that heel plantar sensitivity, visual contrast sensitivity and MMSE were significant determinants, together explained approximately 40% of the variance in FR (Table 3).

**Table 2:** Pearson or Spearman correlation coefficients between balance performance and potential determinants.

	FR	FTSTS	TURN
**Control group**			
**Leg muscle strength**			
Hip flexion	0.006	-0.209	-0.04
Knee extension	0.336*	-0.183	-0.299*
Ankle dorsiflexion	0.127	-0.186	-0.222
Ankle plantarflexion	0.046	-0.156	-0.238
**Health conditions**			
BMI	-0.101	0.087	0.257*
Grip strength	0.092	-0.11	0.053
MMSE	-0.132	-0.052	-0.04
Hypertension	-0.098	0.104	0.064
Cardiac disease	-0.029	0.101	-0.035
Falls	-0.014	0.081	0.011
**Diabetes group**			
**Leg muscle strength**			
Hip flexion	0.133	-0.398**	-0.294**
Knee extension	0.227	-0.557**	-0.516**
Ankle dorsiflexion	0.288*	-0.614**	-0.469**
Ankle plantarflexion	0.145	-0.133	0.168
**Health conditions**			
BMI	0.185	0.167	0.123
Grip strength	0.034	-0.432**	-0.251*
MMSE	0.448**	-0.243*	-0.345*
Hypertension	0.011	0.11	0.022
Cardiac disease	0.064	0.18	-0.091
Falls	0.106	-0.022	-0.112

* p<0.05

** p<0.01

**Table 3:** Results of hierarchical regression analysis.

	Total R^2^	Factors entered	ΔR^2^	*p*	CI	Beta	*p*	VIF
**Control Group**								
**FR**	0.113			0.006				
		Heel sensitivity^#^						
		Visual contrast^#^						
		Knee extension	0.113	0.006	-0.071~0.402	0.336	0.006	1.000
**FTSTS**	NA							
		Heel sensitivity^#^						
		Visual contrast^#^						
**TURN**	0.089			0.014				
		Heel sensitivity^#^						
		Visual contrast^#^						
		Knee extension	0.089	0.014	-6.043~-0.708	-0.299	0.014	1.000
		BMI^#^						
**Diabetes group**								
**FR**	0.390			0.001				
		Heel sensitivity	0.062	0.002	0.005~0.056	0.263	0.039	1.059
		Visual contrast	0.198	0.006	0.004~0.015	0.416	0.001	1.119
		Ankle dorsiflexion^#^						
		Diabetes duration^#^						
		MMSE	0.13	0.006	0.003~0.014	0.372	0.001	1.064
**FTSTS**	0.377			<0.001				
		Heel sensitivity^#^						
		Visual contrast^#^						
		Hip flexion^#^						
		Knee extension^#^						
		Ankle dorsiflexion	0.377	<0.001	-42.137~-22.789	-0.614	<0.001	1.000
		Diabetes duration^#^						
		MMSE^#^						
		Grip^#^						
**TURN**	0.391			0.001				
		Heel sensitivity	0.056	0.038	-0.826~0.006	-0.187	0.1	1.027
		Visual contrast^#^						
		Hip flexion^#^						
		Knee extension	0.242	<0.001	-8.095~ -2.785	-0.397	<0.001	1.131
		Ankle dorsiflexion^#^						
		Diabetes duration^#^						
		MMSE	0.093	0.008	-0.234~ -0.059	-0.32	0.008	1.108

^#^not included in the selected regression model

CI: confidence interval; VIF: variance inflating factors; NA: not available because no factor was selected.

### Five times sit-to-stand

For the control group, FTSTT was not significantly correlated with TURN (r = 0.172, *p* = 0.164) or the lower limb strength or health conditions (Table 2). Hence only two blocks, sensory function and age/gender, were entered in the regression model and found no significant determinant (Table 3). For the diabetes group, FTSTT was significantly correlated with TURN (r = 0.505, *p* <0.001), the strength of hip flexion, knee extension, ankle dorsiflexion and hand grip, and MMSE (Table 2). The results of regression analysis showed that only the ankle dorsiflexion strength was a significant determinant and alone explained 40% of the variance in FTSTT (Table 3).

### Turn 180°

For the control group, TURN was significantly correlated with knee extension strength and BMI (Table 2). The results of the regression analysis showed that knee extension strength was a significant determinant and explained approximately 9% of the variance in FTURN (Table 3). For the diabetes group, TURN was significantly correlated with the strength of hip flexion, knee extension, ankle dorsiflexion and hand grip, and MMSE (Table 2). The results of regression analysis showed that knee extension strength and MMSE were significant determinants, and together with plantar sensitivity, age and gender explained approximately 40% of the variance in TURN (Table 3).

## Discussions

Aging is well accepted to be associated with declines in balance-related functions. Some pathologies and their interactions with aging changes can lead to further balance impairments unique to the patient population. This study found that diabetes in older adults was associated with significant declines in the ability to sit-to-stand and turn180°, but not forward reach. And the primary determinants for these tasks were ankle dorsiflexion strength, knee extension strength, and visual contrast sensitivity, respectively.

Functional Reach measures the ability to move the body’s center of mass forward within a fixed base of support. A shorter reach distance indicates poorer functional balance and has been found to be associated with frailty [23], falling [24] and a number of common health problems, such as stroke, diabetes neuropathy, or Parkinson’s disease in the elderly [25–27]. It has been reported that middle-aged people (mean age = 53 years) with diabetes but not neuropathy did not differ from non-diabetic individuals in reach distance [28]. In this study, one-third of the diabetes participants had neuropathy and the averaged plantar sensitivity and lower limb muscle strength were significantly poorer than the control group. These deficits, unexpectedly, were not accompanied by a significantly shorter reach distance. This study further found that visual contrast sensitivity and cognition had the greatest independent contribution to reach distance for the diabetes group. It seems possible that better visual contrast sensitivity, which could allow the use of visual cues to compensate for impaired plantar sensitivity, and better cognitive function, which could enable the development of movement solutions, could help to offset the lower limb sensorimotor deficits common in the diabetes group.

The task of FTSTS involves changing the base of support between the buttocks and feet repeatedly and places high demands on vision, proprioception, coordination, and especially lower limb strength [29–31]. A longer FTSTS time has been observed in people with balance problems, both young and older [21], and found to predict falls and disability in older adults [21,32]. The performance of FTSTS of middle-aged diabetes patients was found to be poorer than age-matched non-diabetic adults [33]. This study provided evidence to show that such diabetes-related discrepency in performance also existed in older adults.

This study further found that the ankle dorsiflexion strength was the only signifcant determinant for FTSTS. Although repeatedly stanidng up and sitting down is required during this task, it is the former subtask that is challenging. Standing up from a seated position involves a continous body motion of forward rotation and upward displacement, and changing the base of support from the buttocks to the feet. The ankle dorsiflexing torque not only can stabilize the contact between the feet and the ground but also rotate the lower leg forward and help to add to the knee extension torque in bringing the body forward and upward. The ability to generate ankle dorsiflexion torque (i.e ankle dorsiflexion strength) could be particularly important for the diabetes group who had poorer knee extension strength. And since the ankle dorsiflexion strength was limited, the performance of FTSTS also decreased in the diabetes group.

The task of turning 180° involves actions frequently needed in daily living but has received little attention in research. Poorer TURN performance has been found to be associated with a higher risk of falls and loss of independent living in older adults [34,35]. It has been proposed that compared to straight-line walking, turning required more cognitive function for the processing of sensory inputs [36,37]. Furthermore, in older adults without dementia, turning was found to be associated with the ability to compare objects quickly and mentally manipulating 2- or 3-dimensional figures [38]. In this study, MMSE was also found to be an independent and significant determinant for TURN.

The strongest determinant for TURN, however, was knee extension strength. Standing on one leg while moving the rest of the body is the primary subtask for TURN. To maintain upright during single leg standing, the ability to generate sufficient knee extension torque is highly important to counteract the gravitational force. Because the diabetes group had a greater BMI, the demand on the knee extending torque would be higher. Possibly as a result, the contribution of knee extension strength on TURN was higher for the diabetes group. And since both MMSE and knee extension strength were significantly poorer, the TURN performance was decreased in the diabetes group.

It is a common observation that when the task demands are sufficiently lower than one’s maximal functional capacity, the association between the task performance and the functional capacity would be lower. In this study, it is reasonable to assume that the balance tasks were not as challenging for the control group because they had significantly better functional capacity than the diabetes group. In this case, the maximal strength would have small contribution to the balance performance.

The strengths of this study included that it provides information for clinical assessment and intervention planning. The study specifically focused on Chinese older adults because they are among the fastest growing population of diabetes [39] but have not yet been well studied. What is more, this study used three different performance-based balance tests to represent different domains of functional balance abilities, and a multitude of their potential determinants were investigated. This study was a cross-sectional study and the causal effect could not be ascertained. Because the information about comorbidities was obtained from self-report and the prevalence and contribution of this problem might be underestimated.

In conclusion, diabetes in older adults was associated with specific functional balance impairments whose primary underpinning functional limitations differed between tasks and from those of the control group. For the diabetes group, the strongest determinant for FR, FTSTS and TURN was visual contrast, ankle dorsiflexion strength and knee extension strength, respectively. These findings suggested that impairments in functional balance ability in older adults with diabetes may not simply be a more severe form of typical aging changes. They could be specific to the task demands and thus warrant separate examination and intervention.

## Supporting Information

S1 File Questionnaire. Questionnaires used for basic information and medical/health histories and Chinese version of Mini Mental Status Examination.
(PDF)
