# Supplementary material for: Functional Balance and Its Determinants in Older People with Diabetes
Source: PLoS One. 2016 Jul 28;11(7):e0159339. doi: 10.1371/journal.pone.0159339 (PMC4965190; doi:10.1371/journal.pone.0159339)

S1 File. **Questionnaire**.
Questionnaires used for basic information and medical/health histories and Chinese version of Mini Mental Status Examination.

Code:___________ tester:__________ Date:__________

Basic Information Questionnaire

Subject Name:________________ Gender:______ Birth date:_______/_____/_____ Age:_____

Emergency Contact Person

Name:_________________ Relationship:_________ Cell phone number:_______________________

Height:___________cm

Weight:____________Kg

Dominant Hand: Right/Left

Dominant Foot: Right/Left

Onset of DM: ____(year)

Education level:

□Illiterate □Elementary school □Junior high school □Senior high school

□Univesity □Graduate school □Others__________

Years of education：__________ years

Medical and health history

With	Without	Disease	Note
☐	☐	Diabetes mellitus	
☐	☐	Diabetic neuropathy	
☐	☐	Diabetic renopathy	
☐	☐	Diabetic nephropathy	
☐	☐	Foot ulcers	
☐	☐	Parkinson's Disease	
☐	☐	Hypertension	

☐	☐	Stroke	
☐	☐	Cardiovascular disease	
☐	☐	Arthritis	
☐	☐	Any problems affected walking, balance, sensory function	
☐	☐	Fall (within 1 year)	
☐	☐	Lower limb pain back pain last over 3 month (within 1 year)	
☐	☐	Others	

Chinese version

Mini-Mental State Examination (MMSE)

Maximum Score	Patient's Score	Questions
5		"What is the year? Season? Date? Day of the week? Month?"
5		"Where are we now：<br>Country? County/city? Town/ district? Hospital/ Institution ? Floor?"
3		The examiner names three unrelated objects (Glasses, Red, Honest) clearly and slowly, then asks the patient to name all three of them. The patient's response is used for scoring. The examiner repeats them until patient learns all of them, if possible.
2		Please answer the questions below:<br>(1)2+4=_____<br>(2)7-3=_____
5		"I would like you to count backward from 100 by sevens." (93, 86, 79, 72, 65, …) Stop after five answers.<br>Alternative: "Take a walk in the park" (in Chinese)
3		"Earlier I told you the names of three things. Can you tell me what those were?"
2		Show the patient two simple objects (a wristwatch and a pencil), and ask the patient to name them.
1		Repeat a 7-character phrase in Chinese
3		"Take the paper in your right hand, fold it in half, and put it on the floor." (The examiner gives the patient a piece of blank paper.)
1		"Please read this and do what it says." (Written instruction is "Close your eyes.")
1		"Please write down your name."
1		"Make up and write a sentence about anything." (This sentence must contain a noun and a verb.)
1		"Please copy this picture." (The examiner gives the patient a blank piece of paper and asks him/her to draw the symbol below. All 10 angles must be present and two must intersect.)
33		TOTAL

Close your eyes

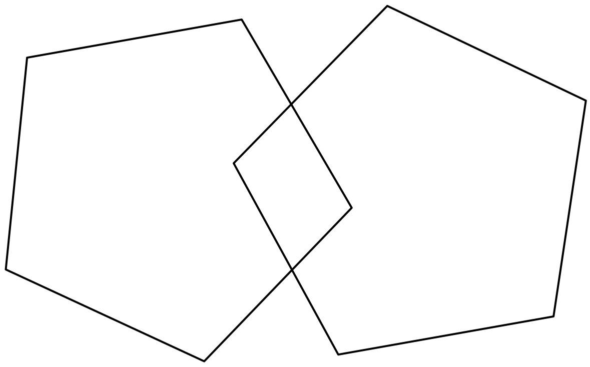

Supplement: S1 File — Questionnaires used for basic information and medical/health histories and Chinese version of Mini Mental Status Examination. (PDF) [file pone.0159339.s001.pdf]
